# miR-125b Promotes Colorectal Cancer Migration and Invasion by Dual-Targeting CFTR and CGN

**DOI:** 10.3390/cancers13225710

**Published:** 2021-11-15

**Authors:** Xiaohui Zhang, Tingyu Li, Ya-Nan Han, Minghui Ge, Pei Wang, Lina Sun, Hao Liu, Tianyu Cao, Yongzhan Nie, Daiming Fan, Hao Guo, Kaichun Wu, Xiaodi Zhao, Yuanyuan Lu

**Affiliations:** 1 State Key Laboratory of Cancer Biology, National Clinical Research Center for Digestive Diseases, Xijing Hospital of Digestive Diseases, Fourth Military Medical University, Xi’an 710032, China; 15829920711@163.com (X.Z.); HYN_87@163.com (Y.-N.H.); hownew@foxmail.com (H.L.); ctyumiao@163.com (T.C.); yongznie@fmmu.edu.cn (Y.N.); fandaim@fmmu.edu.cn (D.F.); kaicwu@fmmu.edu.cn (K.W.); 2 Department of Gastroenterology, National Clinical Research Center for Geriatric Diseases, The Second Medical Center, Chinese PLA General Hospital, Beijing 100853, China; tingyu1990@aliyun.com; 3 Department of Gastroenterology, Xi’an Children’s Hospital, Xi’an 710003, China; sunln13@163.com; 4 State Key Laboratory of Translational Medicine and Innovative Drug Development, Jiangsu Simcere Diagnostics Co., Ltd., Nanjing 210042, China; minghui.ge@simceredx.com (M.G.); h.guo@foxmail.com (H.G.); 5 Department of Gastroenterology, Ningxia Medical University, Yinchuan 750004, China; wangpei_st@163.com

**Keywords:** colorectal cancer, migration, invasion, metastasis, miRNA

## Abstract

**Simple Summary:**

Colorectal cancer (CRC) is the third leading cause for cancer related death, in which metastasis exerts a pivotal role. Therefore, we aim to find out the possible mechanism underlying CRC metastasis. We found that the level of miR-125b was elevated in normal, primary CRC, and distant metastasis tissues stepwise, and high level miR-125b was positively correlated with lymph node metastasis and tumor differentiation. In vitro and in vivo assays showed miR-125b significantly promoted CRC migration and invasion. To elucidate the potential mechanism, cystic fibrosis transmembrane conductance regulator (CFTR) and cingulin (CGN) were defined as two target genes of miR-125b. On the one hand, miR-125b promoted epithelial-mesenchymal transition (EMT) and the production and secretion of urokinase plasminogen activator (uPA) by inhibiting CFTR; on the other hand, miR-125b activated Ras Homolog Family Member A (RhoA)/Rho Kinase (ROCK) signaling by repressing CGN. Therefore, we provided a potential biomarker for CRC prevention and treatment in the future.

**Abstract:**

Metastasis contributes to the poor prognosis of colorectal cancer, the causative factor of which is not fully understood. Previously, we found that miR-125b (Accession number: MIMAT0000423) contributed to cetuximab resistance in colorectal cancer (CRC). In this study, we identified a novel mechanism by which miR-125b enhances metastasis by targeting cystic fibrosis transmembrane conductance regulator (CFTR) and the tight junction-associated adaptor cingulin (CGN) in CRC. We found that miR-125b expression was upregulated in primary CRC tumors and metastatic sites compared with adjacent normal tissues. Overexpression of miR-125b in CRC cells enhanced migration capacity, while knockdown of miR-125b decreased migration and invasion. RNA-sequencing (RNA-seq) and dual-luciferase reporter assays identified CFTR and CGN as the target genes of miR-125b, and the inhibitory impact of CFTR and CGN on metastasis was further verified both in vitro and in vivo. Moreover, we found that miR-125b facilitated the epithelial-mesenchymal transition (EMT) process and the expression and secretion of urokinase plasminogen activator (uPA) by targeting CFTR and enhanced the Ras Homolog Family Member A (RhoA)/Rho Kinase (ROCK) pathway activity by targeting CGN. Together, these findings suggest miR-125b as a key functional molecule in CRC and a promising biomarker for the diagnosis and treatment of CRC.

## 1. Introduction

Colorectal cancer (CRC) is one of the most common malignancies in the world, with up to 1.8 million new cases diagnosed every year [1]. Despite substantial research efforts, CRC ranked third in cancer mortality in 2018, due mainly to the lack of specific symptoms in the early stage of disease, drug resistance, recurrence, economic challenges, and in particular, its high metastatic potential. Hence, it is imperative to discover the underlying mechanisms of metastasis in CRC.

MicroRNAs (miRNAs) are a group of endogenous single-stranded RNA molecules that can prevent protein translation or promote RNA degradation through sequence-specific binding to the 3′ untranslated regions (3′ UTRs) of their target mRNAs [2]. It has been reported that miRNAs function in both physiological and pathological processes in cancer cells by interfering with the expression of critical cancer-related genes [3]. In our previous study, we found that miR-125b is overexpressed in cetuximab-resistant CRC, indicating its crucial role in CRC targeted-drug resistance [4]. Other studies indicated that miR-125b promotes CRC metastasis by targeting MCL1 [5] and enhances 5-FU resistance by activating the APC-mediated Wnt/β-catenin pathway [6]. Conversely, miR-125b was reported as a cancer suppressor in other cancer types, such as breast cancer [7] and cutaneous squamous cell carcinoma [8]. These findings suggest that miR-125b exerts a context-dependent role in cancer development, and it is of particular interest to find novel miR-125b targets that are causally involved in CRC carcinogenesis.

Cystic fibrosis transmembrane conductance regulator (CFTR) is a member of ATP binding cassette (ABC) proteins, containing two transmembrane domains, a regulatory domain and two nuclear binding domains [9]. Unlike other members of this family, CFTR functions as an anion channel for Cl^−^ and HCO_3_^−^, affecting the secretion and absorption of anions and water in epithelial tissues, and CFTR dysfunction results in mucus overproduction and other clinical manifestations [10]. Mutation of CFTR is the underlying cause of cystic fibrosis, one of the most life-threatening genetic diseases in northern European populations, and CFTR modulator therapy has shown remarkable effects in recent years [11]. Furthermore, evidence has also linked patients with cystic fibrosis (CF) to a higher risk of CRC than healthy volunteers [12,13]. Increasing evidence indicates that CFTR is depleted in CRC [14] and functions as a suppressor in CRC metastasis by activating the AF-6-mediated ERK pathway [15]. Conditional knockdown of CFTR promoted CRC carcinogenesis [16]. Moreover, CFTR suppresses the progression of many other cancer types, such as lung cancer [17], breast cancer [18], nasopharyngeal carcinoma [19], and prostate cancer [20]. Nevertheless, the role of CFTR in CRC metastasis is largely unknown and remains to be elucidated.

Cingulin (CGN) is a novel protein localized on the cytoplasmic surface of epithelial tight junctions. CGN forms a parallel dimer of two subunits, each with a globular head, a coiled-coil rod, and a globular tail [21], and CGN functions as a bridge between tight junctions and the cytoskeleton. For example, CGN interacts with several tight junction -related proteins, such as ZO-1, ZO-2 [22], and AF-6 [23], and it was initially discovered by copurification with myosin from intestinal brush border cells [24]. CGN participates in the modulation of the endothelial barrier function in both the human and the mouse brain [25], functions as part of the blood-brain barrier/neurovascular unit [26], and contracts the migratory neural crest cell domain, which is correlated with a strengthening of the neural tube basal lamina [27]. Moreover, CGN compromises RhoA activity, inhibits G1/S phase transition, and prevents cell proliferation in MDCK cells [28]. Thus, CGN possesses essential and versatile functions of modulating endothelial barriers, cell migration and proliferation, but its potential role in CRC metastasis remains unclear.

In our study, we found that miR-125b was elevated in CRC metastatic tissues and that high levels of miR-125b promoted cell migration and invasion. Mechanistically, miR-125b facilitated the epithelial-mesenchymal transition (EMT) process and urokinase plasminogen activator (uPA) secretion by inhibiting CFTR and activated Ras Homolog Family Member A (RhoA)/Rho Kinase (ROCK) signaling by repressing CGN. Therefore, targeting miR-125b may have therapeutic implications for the prevention of CRC metastasis.

## 2. Results

### 2.1. miR-125b Expression Is Upregulated in Metastatic CRC Tissues

**Figure 1:**
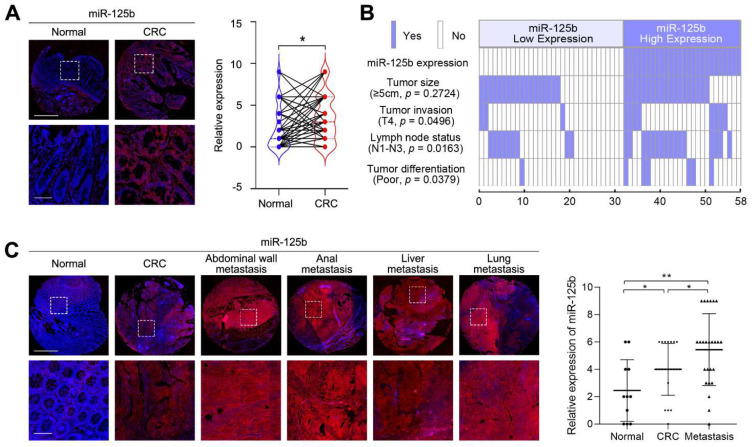
miR-125b expression is upregulated in metastatic colorectal cancer (CRC) tissues. (**A**) Representative images and analysis of fluorescence in situ hybridization (FISH) staining for miR-125b in 58 cases of CRC tissues and their matched adjacent normal tissues. *p* = 0.0130 (*n* = 58; Normal vs. CRC). Magnified views of the regions indicated by the boxed area are shown below. Scale bars represent 200 μm (upper) and 100 μm (lower). (**B**) miR-125b expression positively correlated with tumor invasion, lymph node metastasis and tumor differentiation. (**C**) Representative images and analysis of miR-125b expression in normal colon tissues, primary CRC tissues and metastatic tissues detected by FISH. *p* = 0.0489 (Normal vs. CRC); *p* = 0.0423 (CRC vs. Metastasis); *p* = 0.0025 (Normal vs. Metastasis); *n* (Normal) = 11; *n* (CRC) = 21; *n* (Metastasis) = 25. Scale bars represent 500 μm (upper) and 100 μm (lower). * *p* < 0.05, ** *p* < 0.01 by Student’s *t*-test.

To investigate the relevance of miR-125b in CRC development and progression, we first examined miR-125b expression in a tissue microarray containing 58 CRC tissues and their matched adjacent normal tissues by fluorescence in situ hybridization (FISH). We found that miR-125b levels were increased in 26 (44.8%) CRC tissues compared with their adjacent normal tissues (Figure 1A). Furthermore, correlation analysis showed that high miR-125b expression was significantly associated with a more aggressive CRC phenotype (Table 1). Notably, miR-125b expression was correlated with tumor invasion and lymph node metastasis (Figure 1B), suggesting the involvement of miR-125b in CRC invasion and metastasis. Therefore, we then determined the expression levels of miR-125b in a metastatic CRC tissue microarray containing 11 tumor-adjacent normal tissues, 21 primary CRC tissues and 25 distant metastatic tissues. We found that miR-125b expression levels increased stepwise in primary CRC and distant metastatic tissues compared to adjacent normal tissues (Figure 1C). Collectively, these results indicate that miR-125b is clinically associated with CRC metastasis.

### 2.2. miR-125b Promotes CRC Cell Migration, Invasion and Metastasis

**Figure 2:**
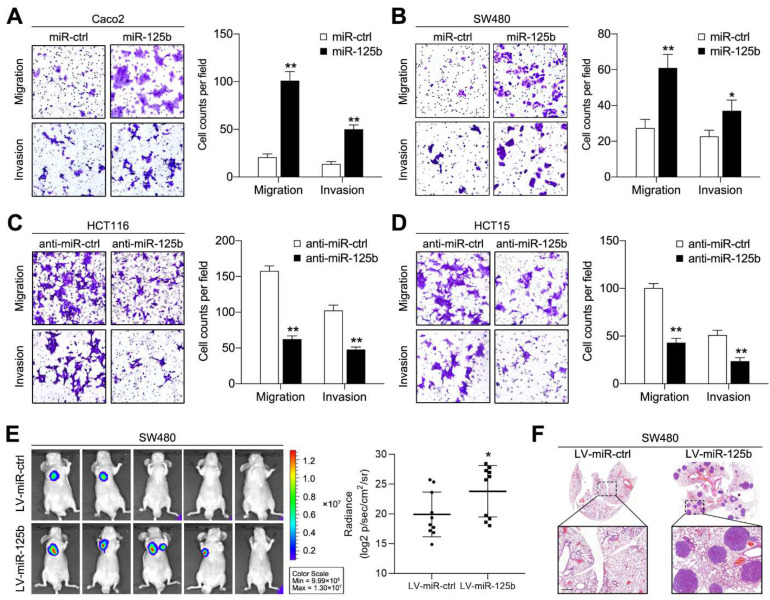
miR-125b promotes CRC cell migration, invasion and metastasis. (**A**,**B**) Transwell migration and invasion assays using Caco2 (**A**) and SW480 (**B**) cells transfected with miR-125b mimic or control mimic (miR-ctrl). (**C**,**D**) Transwell migration and invasion assays using HCT116 (**C**) and HCT15 (**D**) cells transfected with miR-125b inhibitor or control inhibitor (anti-miR-ctrl). Representative images are shown on the left, and quantification of 5 randomly selected fields is shown on the right. (**E**) Representative bioluminescence images of mice (*n* = 10) after tail vein injection of SW480 cells infected with lentiviral miR-125b (LV-miR-125b) or negative control (LV-miR-ctrl). The signal intensity was detected and calculated. (**F**) Representative hematoxylin and eosin (H&E)-stained lung tissue sections of mice from the indicated groups. Scale bars represent 500 μm. * *p* < 0.05, ** *p* < 0.01 by the Student’s *t*-test.

To dissect the exact role of miR-125b in CRC invasion and metastasis, we transfected HCT116 and HCT15 cells (which exhibit high endogenous miR-125b expression) with miR-125b inhibitors and transfected SW480 and Caco2 cells (which exhibit low endogenous miR-125b expression) with miR-125b mimic and verified the infection efficiency by qPCR (Figure S1A). Then, transwell assays were conducted to assess cell migration and invasion. The results showed that miR-125b overexpression enhanced migration and invasion in Caco2 and SW480 cells (Figure 2A,B), whereas miR-125b knockdown markedly inhibited migration and invasion in HCT116 and HCT15 cells (Figure 2C,D). Furthermore, to explore whether miR-125b exerts the same function in vivo, SW480 cells stably expressing miR-125b were injected into nude mice through the tail vein. Upregulation of miR-125b expression contributed to elevated total bioluminescence flux in the lungs compared to that in the control group (Figure 2E). In addition, pathological analysis identified more metastatic foci in the miR-125b-overexpressing group than in the control group (Figure 2F). Together, these findings, collected both in vitro and in vivo, indicate that miR-125b plays an important role in promoting CRC metastasis.

### 2.3. CFTR and CGN Are Direct Targets of miR-125b in CRC

**Figure 3:**
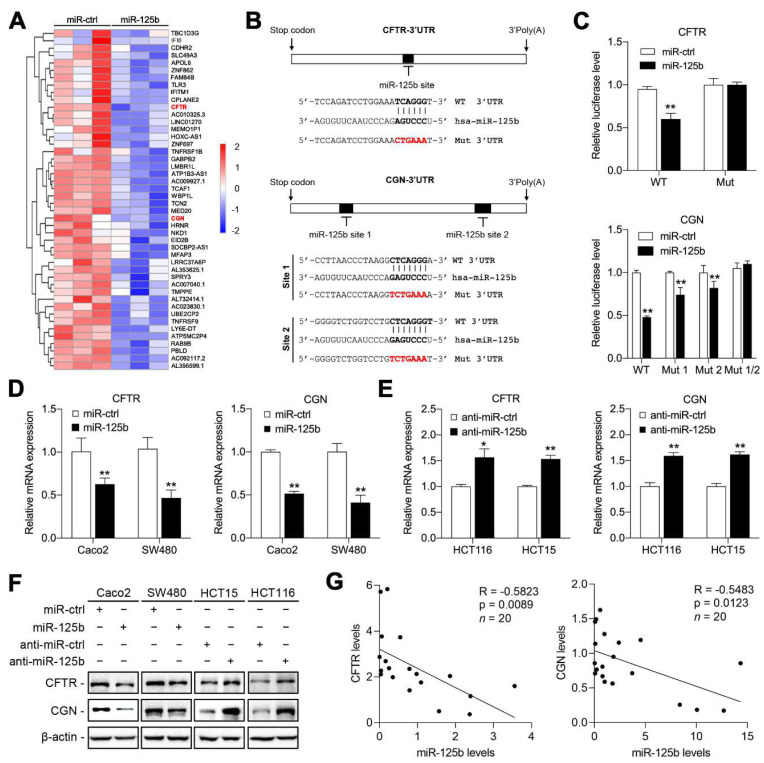
Cystic fibrosis transmembrane conductance regulator (CFTR) and cingulin (CGN) are direct targets of miR-125b. (**A**) Heatmap showing the 46 downregulated genes in Caco2 cells after transfection of miR-125b. The scale bar shows color-coded differential expression from the mean. (**B**) Bioinformatics analysis by TargetScan identified one binding site of miR-125b in CFTR 3′ untranslated region (3′ UTR) and two binding sites in CGN 3′ UTR. (**C**) Relative luciferase activity in SW480 cells cotransfected with wild-type or mutated reporter plasmids and miR-125b or miR-ctrl. (**D**,**E**) qPCR analysis of CFTR and CGN mRNA levels in the indicated CRC cells. (**F**) Western blotting analysis of CFTR and CGN protein levels in the indicated CRC cells. β-actin was used as a loading control. (**G**) Correlation analysis between miR-125b expression and CFTR or CGN mRNA expression in 20 cases of CRC. * *p* < 0.05, ** *p* < 0.01 by the Student’s *t*-test in (**C**–**E**), and by Pearson’s correlation analysis in (**G**).

miRNAs exert their effects by binding the 3′ UTRs of target genes. To identify the specific targets through which miR-125b promotes metastasis, we conducted an RNA sequencing (RNA-seq) analysis of Caco2 cells transfected with miR-125b and the mimic control. A total of 46 downregulated genes were identified (*p* < 0.05; Figure 3A, Table S1). Among them, CFTR is a member of the ABC protein family and acts as an anion channel, facilitating the secretion of chloride and bicarbonate. CFTR serves as a pivotal tumor suppressor through complex mechanisms; CGN, another downregulated gene, is located in the cytoplasmic face of tight junctions and plays an indispensable role in tumor cell polarization, proliferation and migration [21,29,30,31]. Therefore, we selected these genes for further investigation. Bioinformatics analysis by using the TargetScan database identified one binding site for miR-125b in the CFTR 3′ UTR and two binding sites in the CGN 3′ UTR (Figure 3B). To determine whether CFTR and CGN are direct targets of miR-125b, we performed luciferase reporter assays. CFTR and CGN 3′ UTRs containing the miR-125b-binding sites and their mutant counterparts were cloned downstream of the luciferase open reading frame. miR-125b overexpression suppressed the luciferase activities of the CFTR and CGN 3′ UTR reporter constructs, whereas the effect was abolished when one and two mutations were introduced into their seed sequences, respectively (Figure 3C). qPCR analysis further confirmed that the mRNA levels of CFTR and CGN were reduced upon miR-125b upregulation and increased upon miR-125b inhibition (Figure 3D,E). Western blot analysis also verified this result at the protein level (Figure 3F). Moreover, we detected miR-125b, CFTR and CGN expression levels in 20 CRC tissue samples by qPCR. miR-125b expression was found to be inversely correlated with CFTR and CGN expression in these CRC specimens (Figure 3G). These results demonstrated that CFTR and CGN are direct targets of miR-125b in CRC.

### 2.4. CFTR and CGN Inhibited CRC Metastasis In Vitro and In Vivo

**Figure 4:**
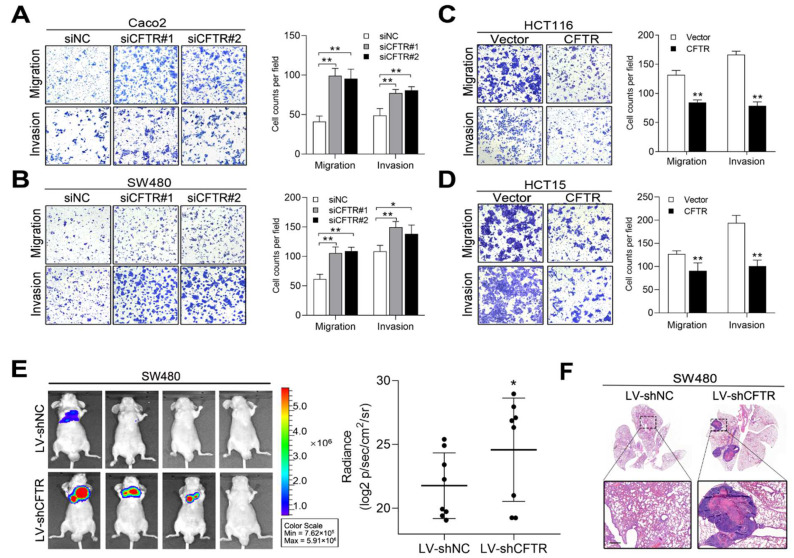
CFTR inhibits CRC metastasis in vitro and in vivo. (**A**,**B**) Silencing CFTR expression by small interfering RNAs (siRNAs) promoted migration and invasion in Caco2 (**A**) and SW480 (**B**) cells. (**C**,**D**) Overexpression of CFTR inhibited migration and invasion in HCT116 (**C**) and HCT15 (**D**) cells. (**E**) Bioluminescent images and radiance values of mice (*n* = 8) injected with SW480 cells infected with lentiviral short hairpin RNA against CFTR (LV-shCFTR) or negative control (LV-shNC). (**F**) Representative H&E staining images of lung tissue sections from the indicated groups. Scale bars represent 500 μm. * *p* < 0.05, ** *p* < 0.01 by the Student’s *t*-test.

**Figure 5:**
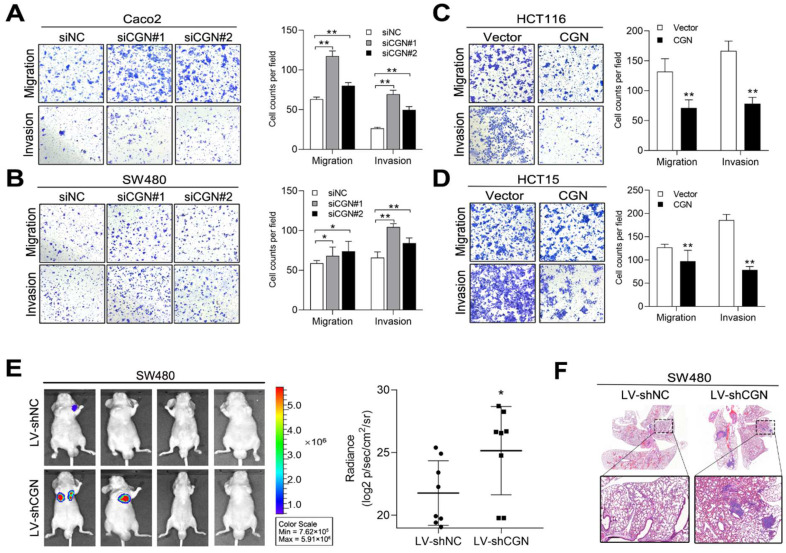
CGN inhibits CRC metastasis in vitro and in vivo. (**A**,**B**) Silencing CGN expression by siRNAs promoted migration and invasion in Caco2 (**A**) and SW480 (**B**) cells. (**C**,**D**) Overexpression of CGN inhibited migration and invasion in HCT116 (**C**) and HCT15 (**D**) cells. (**E**) Bioluminescent images and radiance values of mice (*n* = 8) injected with SW480 cells infected with lentiviral shCGN (LV-shCGN) or negative control (LV-shNC). (**F**) Representative H&E staining images of lung tissue sections from the indicated groups. Scale bars represent 500 μm. * *p* < 0.05, ** *p* < 0.01 by the Student’s *t*-test.

We next investigated whether downregulation of CFTR and CGN would promote CRC cell migration and invasion. We first transiently knocked down CFTR by small interfering RNAs (siRNAs) in Caco2 and SW480 cells (Figure S1B). Transwell assays showed that the downregulation of CFTR increased cell migration and invasion (Figure 4A,B). CFTR-overexpressing HCT116 and HCT15 cells were established by transfection of the CFTR plasmid (Figure S1C). As expected, increased CFTR expression suppressed cell migration and invasion (Figure 4C,D). Furthermore, we performed in vivo metastasis assays by using SW480 cells infected with lentivirus expressing a short hairpin RNA against CFTR (LV-shCFTR) or a negative control (LV-shNC). Six weeks later, lung metastasis was evaluated, and mice in the LV-shCFTR group showed higher total bioluminescence flux in lung metastasis than the control group (Figure 4E). Consistently, metastatic foci in the LV-shCFTR group mice outnumbered those in the control group (Figure 4F). To further test whether CGN modulates the invasion and metastasis of CRC, we constructed CGN-silenced and CGN-overexpressing CRC cell lines, and western blot analysis confirmed the up- and downregulation efficiency (Figure S1D,E). Depletion of CGN promoted migration and invasion in Caco2 and SW480 cells (Figure 5A,B), whereas overexpression of CGN inhibited migration and invasion in HCT116 and HCT15 cells (Figure 5C,D). Similar results were observed in in vivo metastatic assays using SW480 cells infected with LV-shCGN or LV-shNC (Figure 5E,F). Collectively, these results indicated that CFTR and CGN inhibit cell invasion and metastasis, suggesting that they are functional targets of miR-125b in CRC cells.

### 2.5. miR-125b Induces uPA Expression and EMT by Inhibiting CFTR

**Figure 6:**
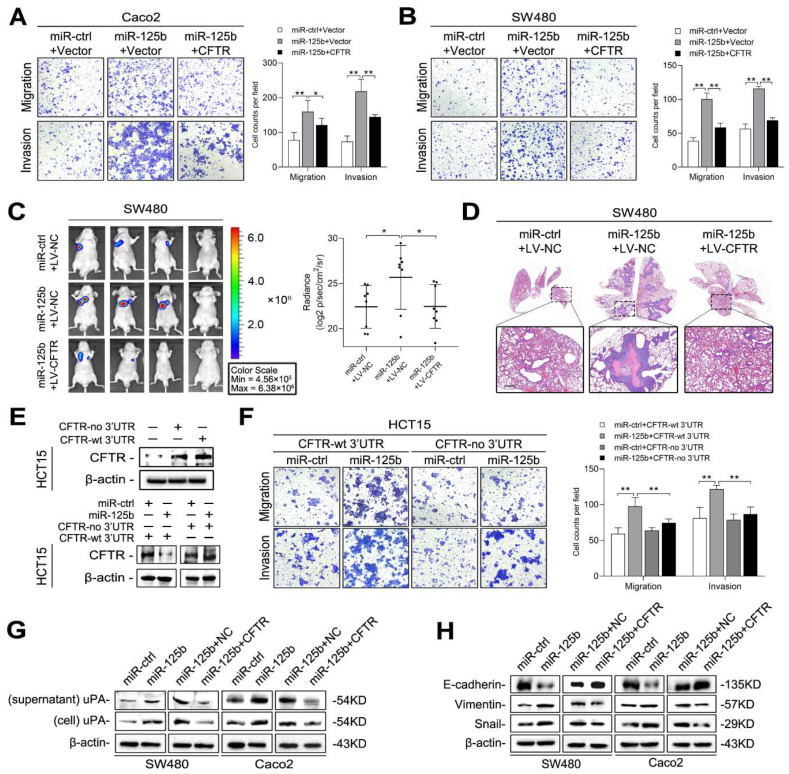
miR-125b induces epithelial-mesenchymal transition (EMT) and urokinase plasminogen activator (uPA) secretion by targeting CFTR. (**A**,**B**) Transwell assays of Caco2 (**A**) and SW480 (**B**) cells cotransfected with miR-125b or miR-ctrl and the CFTR plasmid or control vector. (**C**) Bioluminescent images and radiance values of mice (*n* = 8) injected with SW480 cells after the indicated transduction. (**D**) Representative H&E staining images of lung tissue sections from the indicated groups. Scale bars represent 500 μm. (**E**) CFTR plasmid containing either with or without 3′ UTR was transfected into HCT15 cells, and western blotting was performed 48 h after transfection (upper). HCT15 cells were transfected with CFTR plasmid containing wild-type 3′ UTR or lacking 3′ UTR along with miR-125b or vector control (lower). (**F**) Migration and invasion assays of HCT15 cells transfected with the CFTR expression plasmid with or without 3′ UTR along with miR-125b or miR-ctrl. (**G**) Western blotting analysis of uPA in the indicated cells. (**H**) Western blotting analysis of EMT markers in the indicated cells. * *p* < 0.05, ** *p* < 0.01 by one-way ANOVA followed by Dunnett’s multiple comparison.

To determine whether CFTR is involved in miR-125b-mediated CRC metastasis, we transfected Caco2 and SW480 cells with miR-125b mimic and the CFTR plasmid or the corresponding negative controls. The results indicated that overexpression of CFTR abolished the enhanced cell migration and invasion mediated by miR-125b (Figure 6A,B). We further established SW480 cells that stably expressed miR-125b and/or CFTR and performed in vivo metastasis assays. The increases in luminescence intensity and metastatic foci in the lungs mediated by miR-125b were hindered by overexpression of CFTR (Figure 6C,D). Moreover, we transfected HCT15 cells with a CFTR plasmid with or without its 3′ UTR; when miR-125b was cotransfected into these cells, CFTR expression was markedly reduced in the cells transfected with the plasmid containing the wild-type 3′ UTR but not in cells transfected with the plasmid lacking the 3′ UTR (Figure 6E). Consistently, transwell assays showed that miR-125b could reverse the reductions in cell migration and invasion mediated by the CFTR plasmid with the 3′ UTR, but this effect was abolished by transfection of the CFTR plasmid lacking the 3′ UTR (Figure 6F). These results indicated that CFTR is functionally involved in miR-125b-mediated CRC metastasis.

We then investigated the mechanism by which CFTR regulates CRC cell migration and metastasis. CFTR was reported to inhibit tumorigenesis by suppressing uPA expression [17,18,20]. Since the binding of uPA to its specific membrane receptor, uPAR, initiates a proteolytic cascade that degrades the extracellular matrix (ECM), facilitating cell invasion and metastasis [32], we questioned whether miR-125b promotes metastasis by restoring CFTR-suppressed uPA expression. Western blot analysis indicated that miR-125b overexpression increased uPA expression in both the cell lysate and supernatant, but this effect was mitigated by the restoration of the CFTR expression (Figure 6G). In addition, CFTR has been reported to inhibit the EMT process [16,18,19]. Hence, we examined whether EMT is involved in the metastatic process driven by miR-125b. The results showed that miR-125b overexpression decreased E-cadherin expression but increased the expression of Vimentin and Snail. More importantly, restoration of CFTR abolished the effect of miR-125b on expression of these EMT markers (Figure 6H). These results suggested that miR-125b promotes uPA expression and EMT by inhibiting CFTR in CRC cells.

### 2.6. miR-125b Activates RhoA/ROCK Signaling by Targeting CGN

**Figure 7:**
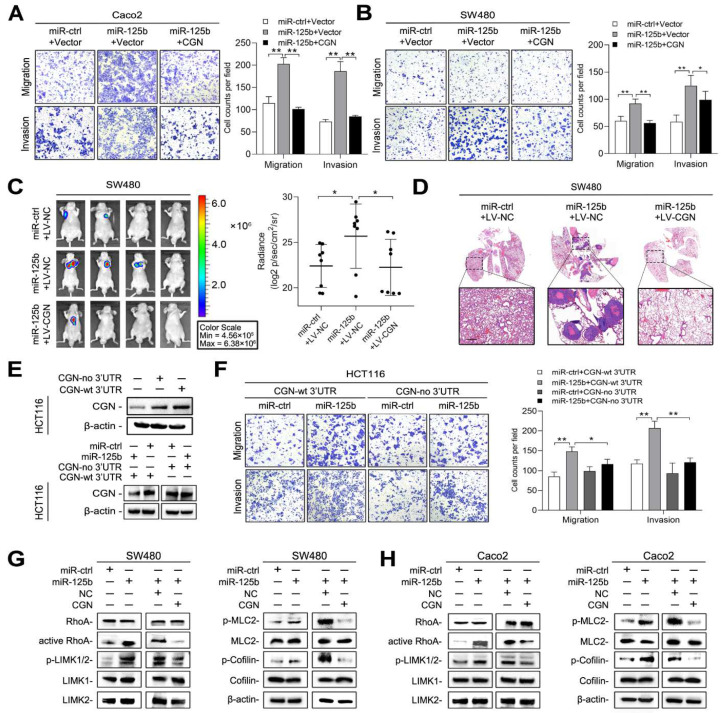
miR-125b activates Ras Homolog Family Member A (RhoA)/Rho Kinase (ROCK) signaling by targeting CGN. (**A**,**B**) Transwell assays of Caco2 (**A**) and SW480 (**B**) cells transfected with miR-125b or miR-ctrl and the CGN plasmid or control vector. (**C**) Bioluminescent images and radiance values of mice (*n* = 8) injected with SW480 cells after the indicated transduction. (**D**) Representative H&E staining images of lung tissue sections from the indicated groups. Scale bars represent 500 μm. (**E**) CGN plasmid containing either with or without 3′ UTR was transfected into HCT116 cells, and western blotting was performed 48 h after transfection (upper). HCT116 cells were transfected with CGN plasmid containing wild-type 3′ UTR or lacking 3′ UTR along with miR-125b or vector control (lower). (**F**) Migration and invasion assays of HCT116 cells transfected with the CGN expression plasmid with or without 3′ UTR along with miR-125b or miR-ctrl. (**G**,**H**) Western blotting analysis of proteins in RhoA/ROCK pathway in SW480 (**G**) and Caco2 (**H**) cells after indicated transduction. * *p* < 0.05, ** *p* < 0.01 by one-way ANOVA followed by Dunnett’s multiple comparison.

To determine whether CGN is also involved in miR-125b-mediated metastasis, we transfected CRC cells with miR-125b mimic and CGN plasmid or the corresponding negative controls. Increased CGN expression substantially inhibited the increases in cell migration and invasion induced by miR-125b in both Caco2 and SW480 cells (Figure 7A,B). In vivo metastasis assays further demonstrated that CGN abrogated miR-125b-induced lung metastasis in SW480 cells (Figure 7C,D). We constructed CGN-expressing plasmids with or without its 3′ UTR and found that miR-125b suppressed the expression of only CGN with its 3′ UTR (Figure 7E). Transwell assays further showed that miR-125b reversed the reductions in cell migration and invasion mediated by the CGN plasmid with the 3′ UTR but not the plasmid lacking the 3′ UTR (Figure 7F). These results indicated that CGN is functionally involved in miR-125b-mediated CRC metastasis.

CGN is a critical tight junction-related protein and was reported to influence RhoA activity [28]. Since RhoA/ROCK signaling plays an important role in regulation of the actin cytoskeleton [33], we hypothesized that miR-125b regulates cell migration by targeting CGN-RhoA/ROCK signaling. We found that miR-125b activated the RhoA protein and increased the phosphorylation of LIMK-1/2 and cofilin (Figure 7G,H), which led to a reduction in actin depolymerization [34]. miR-125b also enhanced the phosphorylation of MLC (Figure 7G,H), which led to an increase in actomyosin contractility [35]. These results suggested that miR-125b activates RhoA/ROCK signaling by targeting CGN in CRC cells.

### 2.7. miR-125b Was Negatively Correlated with CFTR and CGN in CRC Specimens

**Figure 8:**
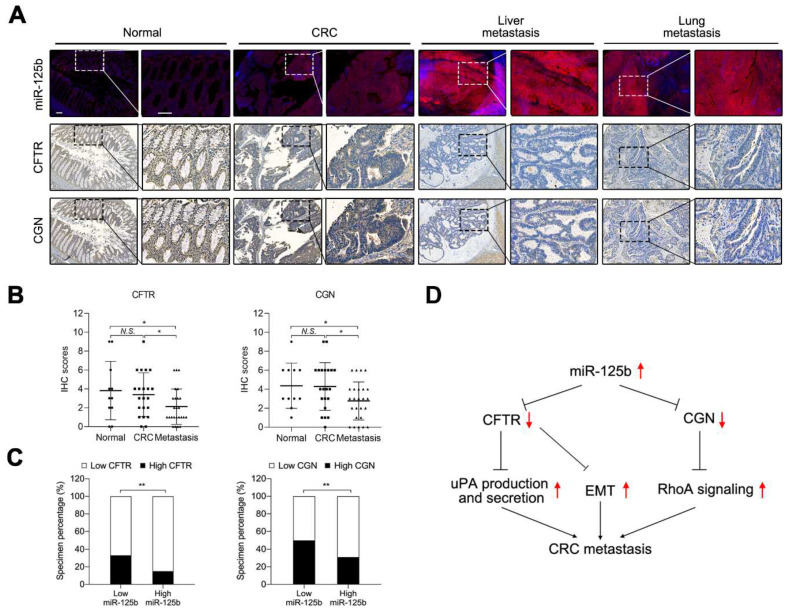
miR-125b is negatively correlated with CFTR and CGN in CRC. (**A**) Representative images of serial sections indicate an inverse relationship between miR-125b and both CFTR and CGN. Magnified views of the regions indicated by the boxed area are shown below. Scale bars represent 200 μm (up) and 100 μm (down). (**B**) CFTR and CGN levels were decreased in metastasis compared with normal and CRC tissues. Left: *p* = 0.6545 (Normal vs. CRC); *p* = 0.0494 (Normal vs. Metastasis) *p* = 0.0472 (CRC vs. Metastasis); Right: *p* = 0.9330 (Normal vs. CRC); *p* = 0.0443 (Normal vs. Metastasis) *p* = 0.0269 (CRC vs. Metastasis). (**C**) The association between miR-125b expression and CFTR and CGN levels in CRC specimens. * *p* < 0.05, ** *p* < 0.01 by Wilcoxon signed rank test in (**B**) and by Chi-square test in (**C**). (**D**) A picture summary of our results.

To examine whether miR-125b targets CFTR and CGN in human CRC, we analyzed the expression patterns of CFTR and CGN in the tissue microarray containing tumor-adjacent normal tissues, primary CRC tissues and distant metastatic tissues. IHC analysis showed that the expression of both CFTR and CGN was frequently decreased in metastatic tissues compared with normal tissues and CRC tissues (Figure 8A,B). miR-125b was found to be inversely related with both CFTR and CGN (Figure 8C). These results suggested that miR-125b was negatively correlated with CFTR and CGN in the human CRC specimens.

Collectively, miR-125b was upregulated in primary CRC and distant metastasis tissues and promoted CRC metastasis both in vitro and in vivo. CFTR and CGN were defined as two target genes of miR-125b. On the one hand, miR-125b promoted EMT and the production and secretion of uPA by inhibiting CFTR; on the other hand, miR-125b activated RhoA/ROCK signaling by repressing CGN. We discovered the potential mechanisms of miR-125b to promote CRC metastasis, and miR-125b could be a novel biomarker for CRC diagnosis and treatment (Figure 8D).

## 3. Discussion

miRNAs are critical regulatory factors in multiple tumors. A review in 2021 summarized the role of various miRNAs such as miR-143, miR-200, miR-155 etc. in colorectal carcinogenesis including proliferation, metastasis, and drug resistance [36]. A previous study from our group showed that miR-125b overexpression was observed in cetuximab-resistant CRC and that miR-125b promotes cetuximab resistance by stimulating the Wnt pathway [4]. Herein, we subsequently investigated whether miR-125b also impacts metastasis in CRC and the underlying mechanisms. Recently, evidence linking miR-125b to CRC has emerged. For instance, by combining differential expression analysis and weighted gene co-expression network analysis, miR-125b was revealed as one of the hub miRNAs related to prognosis in CRC [37]. The combination of miR-125b and 4 other miRNAs in serum exhibited high diagnostic value for CRC, with a sensitivity of 84.7% and a specificity of 98.7% [38]. miR-125b expression was considerably upregulated in metastatic CRC tissues compared with their nonmetastatic counterparts [39]. MIR100HG, a long non- coding RNA (lncRNA) embedded with miR-100 and miR-125b, was found to be increased in CRC tissues compared with paired normal tissues and to promote metastatic behavior in CRC cells, but the underlying mechanism remains largely unknown [37]. Granulocyte colony-stimulating factor (G-CSF), which is increased in CRC tissues, enhanced the total concentration of miR-125b in CRC. Although further research discovered that miR-125b accelerated CRC metastasis by decreasing MCL1 levels, it is still not clear how MCL1 orchestrates the metastatic process [5]. In our study, we proved that miR-125b expression was upregulated in primary CRC and metastatic tissues in a stepwise manner. Correlation analysis revealed that a high level of miR-125b expression in CRC tissues was notably associated with more aggressive tumor hallmarks. Then, we demonstrated its pro-metastatic effect in 4 CRC cell lines by increasing or decreasing miR-125b expression both in vitro and in vivo. Since metastasis and drug resistance are two major causes for cancer morbidity and mortality, we believe that miR-125b is a promising biomarker for the diagnosis and treatment of colorectal cancer.

Mutation of CFTR has been widely recognized as a casual factor for CF. The fact that miR-125b could be a prognostic marker for CF patients also implies that interactions between miR-125b and CFTR could influence CF progression [40]. Several lines of evidence have indicated that CFTR acts as a tumor suppressor in multiple disease scenarios due to its anion channel function. For example, CFTR-mutant Apc^min/+^ mice developed substantially more tumors in the colon and the entire small intestine, with shortened overall survival time [16]. CFTR could accelerate cutaneous wound healing by suppressing the MAPK/NF-κB pathway to relieve inflammation, reduce proliferation and promote the differentiation of keratinocytes [41]. Another study showed that the aggressive phenotype of CRC cells resulting from the lack of CFTR expression could be partially restored by AF-6, a novel intracellular adherent junction protein that interacts with the cytoplasmic region of nectins [15,42]. CFTR also suppresses metastasis in non-small cell lung cancer [17], nasopharyngeal carcinoma [19] and breast cancer [18]. In our study, CFTR was verified as a target gene of miR-125b by RNA-Seq and bioinformatics analysis, luciferase reporter assays, and in vitro and in vivo metastasis assays. CFTR inhibited CRC migration and invasion and was reduced in metastatic tissues compared with primary CRC tumors. Moreover, CFTR was capable of attenuating CRC migration promoted by miR-125b through suppressing EMT and uPA expression.

CGN was originally reported as an acidic, heat-stable protein located in the apical zone of the terminal web, with the putative structure as a parallel dimer with globular heads, coiled-coil rods and globular tails [21]. CGN may bind to noncentrosomal microtubules (MTs), which have been implicated in tight junctions, and knockdown of CGN transformed spherical cell colonies into anisotropic colonies [43]. Moreover, CGN could compromise thrombin-induced endothelial cell permeability and cytoskeletal remodeling through the GEF-H1/RhoA pathway [44]. Lack of CGN suppressed the gut epithelial barrier, and it is crucial for the initiation and progression of irritable bowel syndrome, a functional GI disorder without specific biomarkers [45]. Epithelial tight junctions formed when epithelial cells reached confluence, contributing to the upregulation of CGN expression, downregulation of RhoA expression, and inhibition of G1/S phase transition, which was associated with cell proliferation [46]. However, how CGN functions in CRC metastasis and the mechanism remain elusive. In our work, CGN was primarily identified as a target gene of miR-125b. Functional assays verified CGN as a metastasis inhibitor both in vitro and in vivo. In a human CRC tissue microarray, CGN was found to be decreased in distant metastases compared with primary lesions. Mechanistically, we found that miR-125b could activate RhoA and subsequent RhoA/ROCK signaling, while CGN overexpression impeded this process, providing an intrinsic pathway whereby miR-125b promoted metastasis.

In summary, our findings highlighted the pivotal role of miR-125b in CRC metastasis. Our results indicated that CFTR and CGN are two direct and functional target genes of miR-125b in metastasis. We found that the EMT process and uPA secretion were responsible for CFTR-mediated metastasis inhibition, while the RhoA/ROCK pathway was responsible for CGN-mediated inhibition of metastasis. This study may deepen our understanding of the functional role of miR-125b in CRC metastasis, indicating that it is a promising potential therapeutic target for the treatment of CRC.

## 4. Materials and Methods

### 4.1. Cell Culture

The human CRC cell lines SW480, Caco2, HCT15 and HCT116 were obtained from the American Type Culture Collection (ATCC). All cells were cultured in Dulbecco’s modified Eagle’s medium (DMEM, Thermo Fisher Scientific Gibco, Beijing, China) supplemented with 10% fetal calf serum (Thermo Fisher Scientific Gibco, Beijing, China) and 1% antibiotic-antimycotic (Thermo Fisher Scientific Gibco, Beijing, China) at 37 °C in 5% CO_2_.

### 4.2. Tissue Collection and Tissue Microarrays

Two types of human tissue microarray chips were purchased from Outdo Biotech (Shanghai, China): one containing normal intestinal epithelial tissues and paired CRC tissues (Table S2) and the other containing adjacent normal tissues, primary CRC tissues, and distant metastasis tissues (Table S3). Another 20 paired CRC tissues were collected from patients who had undergone surgical resection at Xijing Hospital, Xi’an, China. This study was approved by the Protection of Human Subjects Committee of Xijing Hospital. Informed consent was obtained from each patient.

### 4.3. Protein Extraction and Western Blot Analysis

Fresh cells were lysed with RIPA lysis buffer supplemented with a complete protease inhibitor cocktail (Roche, Manheim, Germany) and phosphatase inhibitor (Roche, Manheim, Germany) for 5 min on ice. After centrifuged (12,000 rpm, 4 °C, 15 min), the supernatant of cell lysates was collected and denatured by 5× loading buffer at 100 °C for 10 min. A Pierce BCA Protein Assay Kit (Beyotime, Shanghai, China) was used to determine the protein concentration. Approximately 20–30 μg of total protein purified from cell lysates was loaded into the wells of an sodium dodecyl sulfate polyacrylamide (SDS-PAGE) gel (Bio-Rad, Hercules, CA, USA) and subjected to electrophoresis following the manufacturer’s instructions. After transferring the protein from the gel to a membrane and blocking with 10% fat-free milk, we incubated the membrane with primary antibody overnight at 4 °C and scanned the blots by using a Molecular Imager ChemiDox XRS + Imaging System with Image Lab software (Bio-Rad). The primary antibodies were against CFTR (R&D Systems, Minneapolis, MN, USA), CGN (Proteintech, Wuhan, China), Vimentin (CST, Boston, MA, USA), SNAIL (CST), MLC2 (Protientech), p-MLC2 (CST), LIMK1 (Protientech), LIMK2 (Protientech), p-LIMK1/2 (CST), cofilin (Protientech), p-cofilin (CST), and β-actin (CST). Experiments were repeated independently at least three times. (The whole western blots figures see Figure S2).

### 4.4. RNA Extraction and Real-Time PCR

Total RNA was extracted from fresh cells and human tissues by a miRNeasy kit (QIAGEN, Dusseldorf, Germany) according to the manufacturer’s instructions, and purity was determined using A260/A280 spectrophotometric readings. cDNA was synthesized using a PrimeScript RT reagent kit (TaKaRa, Dalian, China). qPCR was performed with SYBR Premix Ex Taq II (TaKaRa) according to the manufacturer’s instructions. The PCR amplification was performed on a CFX96 Real-Time PCR Detection System (Bio-Rad) with the following cycling conditions: 95 °C for 30 s, 40 cycles at 95 °C each for 5 s, 60 °C for 30 s, and a final extension at 95 °C for 10 s, 65 °C for 5 s and 95 °C for 5 s. Primers specific for miR-125b and U6 were designed and synthesized by RiboBio (Guangzhou, China), while primers specific for CFTR, CGN, and β-actin were designed and synthesized by TaKaRa. The housekeeping genes β-actin and U6 were used as internal controls. The PCR primers we used were as follows: CFTR forward, 5′-AACAAGCATTTGCTGATTGCAC-3′; CFTR reverse, 5′-CGTTCAGCAGTTTCTGGATGG-3′; CGN forward, 5′-GAAGCGTTTGCTGGACAGGAC-3′; CGN reverse, 5′-TGCTGCAGGGCTTGCTTAGA-3′; β-actin forward, 5′-TGGCACCCAGCACAATGAA-3′; and β-actin reverse, 5′-CTAAGTCATAGTCCGCCTAGAAGCA-3′. Experiments were repeated independently at least three times.

### 4.5. FISH

FISH assays were performed as described previously [47]. miR-125b probes labeled with digoxigenin (DIG) were synthesized by Exiqon (Copenhagen, Denmark). In addition, nuclei were stained with Hoechst 33342. After mounting with mounting medium (Vector Laboratories, Burlington graham, CA, USA), the slides were scanned, and images were taken by a Nikon ECLIPSE Ti confocal microscope. The data analysis was conducted as previously described [48].

### 4.6. Immunohistochemistry (IHC)

Tissue sections were deparaffinized in xylene for 15 min twice and over a gradient of 100%–75% ethanol solutions, each for 5 min and subjected to antigen retrieval by citrate buffer (pH = 6.0). A 3% H_2_O_2_ solution was used to block endogenous peroxidase activity for 15 min, and then the slides were incubated with primary antibodies against CFTR (R&D systems) and CGN (Proteintech) overnight at 4 °C according to the instructions. After incubation with HRP-conjugated goat anti-rabbit or goat anti-mouse secondary antibody (ZSGB, Beijing, China) for 30 min and staining with 3,3-diaminobenzidine tetrahydrochloride plus (DAB+), the tissue sections were dehydrated in a gradient of 75–100% ethanol solutions, each for 2 min, and xylene for 5 min twice. Finally, they were mounted by mounting medium (ZSGB), scanned by 3D HISTECH and scored by two independent observers. The IHC score was determined by both the staining intensity and staining extent of the molecule of interest in each specimen. The staining intensity was scored as follows: 0, no staining; 1, weak staining; 2, moderate staining; and 3, strong staining. The staining extent was scored based on the percentage of positively stained tumor cells as follows: 0, no positive cells; 1, <10%; 2, 10–50%; and 3, >50%.

### 4.7. Transwell Invasion and Migration Assay

For the invasion and migration assays, chambers with 8-µm-diameter micropore membranes coated with Matrigel (invasion assay) or without Matrigel (migration assay) were inserted into 24-well plates (Corning, Corning, NY, USA). Up to 2 × 10^5^ cells resuspended in 200 µL serum-free medium in different groups were seeded into the upper compartments, and the bottom compartment was filled with 700 µL medium containing 10% fetal calf serum. At a time 48 to 96 h later, invasive cells were fixed with anhydrous ethanol for 5 min and stained with 0.1% crystal violet for 5 min, and the cells remaining in the upper chamber were gently removed with a cotton swab. For each compartment, 5 randomly selected fields under a microscope (Olympus, Tokyo, Japan) were used for further analysis.

### 4.8. In Vivo Metastasis Assay

Nude mice aged between 6 to 8 weeks were obtained from the Experimental Animal Center of the Fourth Military Medical University and were housed in specific pathogen-free conditions. A total of 2 × 10^6^ SW480 CRC cells in mid-log phase were suspended in 200 µL PBS and injected into the nude mice through the tail vein. Six weeks later, we gave these mice a 200 µL intraperitoneal injection of VivoGlo™ Luciferin (Promega, Madison, WI, USA). Then the bioluminescence intensity was detected by using the IVIS Spectrum in vivo imaging system (Perkin Elmer, Shanghai, China), immediately after isoflurane inhalation anesthesia. The mice were euthanized humanely. Lung tissues were fixed in 4% formalin, and hematoxylin and eosin (H&E) staining was performed to count the metastatic foci. The study was approved by the Fourth Military Medical University Animal Care Committee.

### 4.9. Dual-Luciferase Reporter Assay

For the dual-luciferase reporter assay, cells seeded in the 24-well plates were co-transfected with 0.02 nmol miR-ctrl or miR-125b and 0.5 µg psiCHECK^TM^-2 CFTR/CGN vectors with the wild-type or mutant 3′ UTR by using jetPRIME (Polyplus-transfection, New York, NY, USA). The miRNA 3′ UTR luciferase reporter vectors were constructed as previously described [49]. Twenty-four hours after transfection, the cells were harvested, and firefly and Renilla luciferase activities were measured using the dual-luciferase reporter assay (Promega).

### 4.10. Constructs, Oligonucleotides, Infection and Transfection

The miR-125b mimic, inhibitor and corresponding control were synthesized and purified by RiboBio. siRNAs targeting CGN and CFTR were designed and synthesized by GenePharma (Shanghai, China). GV219 vectors expressing CFTR and CGN were purchased from GeneChem (Shanghai, China). RNA and protein were extracted 48 or 72 h after transient infection. Both siRNAs and plasmids were transfected by jetPRIME Transfection Reagent following the manufacturer’s instructions. To generate stable cell lines, miR-125b-overexpressing lentivirus with EGFP-puromycin-FLAG was generated with GV369 vectors by GeneChem. CFTR- and CGN-expressing lentiviruses with luciferase-puromycin-FLAG were produced by GV260 vectors. Lentiviral vectors containing shRNA sequences were constructed using the GV493 vector gcGFP-puromycin-FLAG and designated LV-shCFTR, LV-shCGN, and LV-shNC. The lentivirus luciferase-neomycin-FLAG was produced with GV542 vectors. The volume of virus used for infection was calculated according to the following formula: volume = MOI × the cells counts/the virus titer. MOI was set as 20, and the virus titer was 1 × 10^7^–1 × 10^9^ TU/mL according to the manufacture’s instruction. SW480 cells were infected with lentivirus in a solution containing 5 µg/mL polybrene and selected with 2 µg/mL puromycin or 200 µg/mL G418 for 4 weeks.

### 4.11. RhoA Activity Assay

RhoA activity assay was conducted by using an Active Rho Detection Kit (CST). To harvest cells under nondenaturing conditions, cells cultured in 75 cm^2^ flasks were lysed with 0.5 mL ice-cold lysis/binding/wash buffer plus 1 mM phenylmethylsulfonyl fluoride (PMSF) and transferred to an appropriate tube. After microcentrifugation at 16,000× *g* at 4 °C for 15 min, the supernatant was collected as the cell lysate, and the protein concentration was determined using a BCA protein assay (Beyotime). Then, we mixed the sample, binding protein, and glutathione resin in the spin column and incubated this system at 4 °C to allow GTP-bound GTPase binding to the glutathione resin through GST-linked binding protein. After removing unbound proteins by centrifugation, glutathione resin-bound GTPase was eluted with SDS buffer and analyzed by western blot. Experiments were repeated independently at least three times.

### 4.12. Statistical Analysis

The data were obtained from 3 or more independent experiments. All analyses were performed by GraphPad Prism 8.0, and data are presented as the means ± the standard errors of the means. Differences between the groups were assessed by the Student’s *t*-test, Chi-square test, or nonparametric signed-rank test according to the type of experiment. *p*-values below 0.05 were considered statistically significant.

## 5. Conclusions

While there is a wealth of research on the causes of CRC metastasis, we have yet to fully understand the underlying mechanism. In this study, we demonstrated the metastasis promotor function of miR-125b in CRC cells. CFTR and CGN were identified as two target genes of miR-125b, through which miR-125b promoted CRC metastasis. In addition, a high level miR-125b was associated with lymph node metastasis and poor tumor differentiation, and the inverse relationship between miR-125b and CFTR/CGN was observed in both primary CRC and distant metastasis tissues. Thus, the targeting of miR-125b might represent promising opportunities to improve current CRC therapy.

## Figures and Tables

**Table 1:** Correlation of miR-125b expression with patient clinicopathologic variables in CRC tissues.

Variables	MiR-125b Expression			*p* -Value
	All (*n* = 58)	Low (*n* = 32)	High (*n* = 26)	
Age (years)				>0.9999
≤65	28	15	13	
>65	30	17	13	
Sex				0.7976
male	30	16	14	
female	28	16	12	
Tumor location				0.3629
ascending colon	19	10	9	
transverse colon	9	5	4	
descending colon	7	6	1	
sigmoid colon	23	11	12	
Tumor size (cm)				0.2724
<5	21	14	7	
≥5	37	18	19	
Tumor invasion				0.0457
T1	0	0	0	
T2	3	3	0	
T3	44	26	18	
T4	11	3	8	
Lymph node metastasis				0.021
N0	33	23	10	
N1	19	8	11	
N2	6	1	5	
N3	0	0	0	
AJCC stage				0.0207
stage 1	3	3	0	
stage 2	30	20	10	
stage 3	25	9	16	
Tumor Differentiation				0.0453
well	11	8	3	
moderate	40	23	17	
poor	7	1	6	

## Data Availability

Further information and requests for resources and reagents should be directed to and will be fulfilled by Xiaodi Zhao (leedyzhao@fmmu.edu.cn). DNA constructs and other research reagents generated by the authors will be distributed upon request to other research investigators under a Material Transfer Agreement.

## Supplementary Materials

The following are available online at https://www.mdpi.com/article/10.3390/cancers13225710/s1, Figure S1: Validation of miRNA and protein expression in the indicated cells, Figure S2: The whole Western blots figures, Table S1: Significantly downregulated genes after miR-125b overexpression, Table S2: Clinic-pathological characteristics of patients in Tissue array 1, Table S3: Clinic-pathological characteristics of patients in Tissue Array 2
