# Supplementary material for: miR-125b Promotes Colorectal Cancer Migration and Invasion by Dual-Targeting CFTR and CGN
Source: Cancers (Basel). 2021 Nov 15;13(22):5710. doi: 10.3390/cancers13225710 (PMC8616371; doi:10.3390/cancers13225710)

Figure 3F

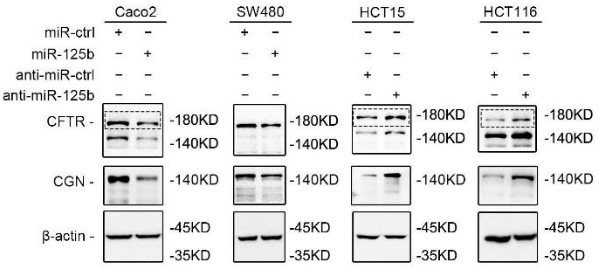

Figure 6E

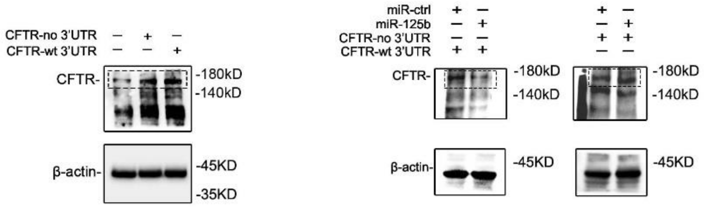

Figure 6G

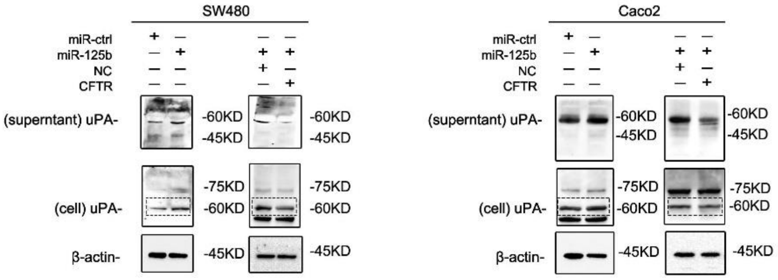

Figure 6H

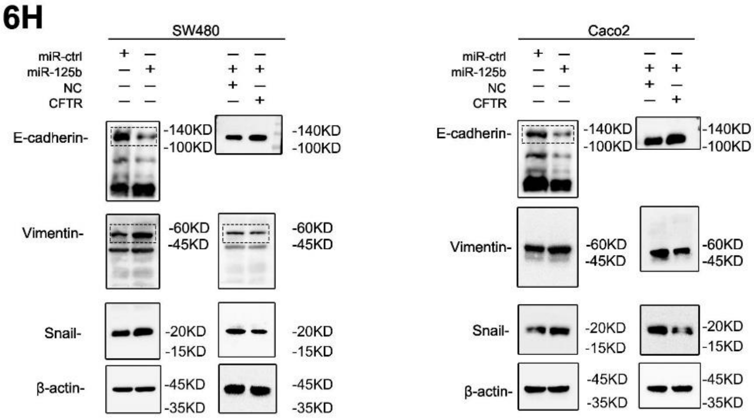

Figure 7E

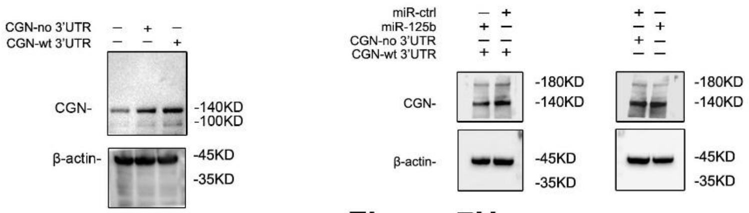

Figure 7G

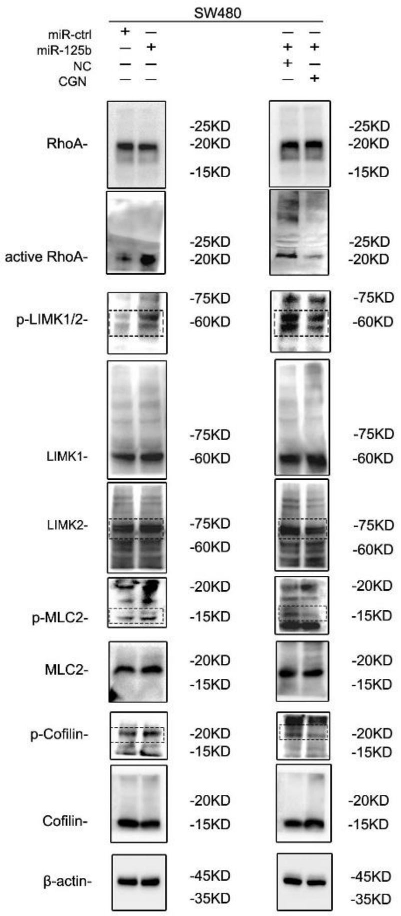

Figure 7H

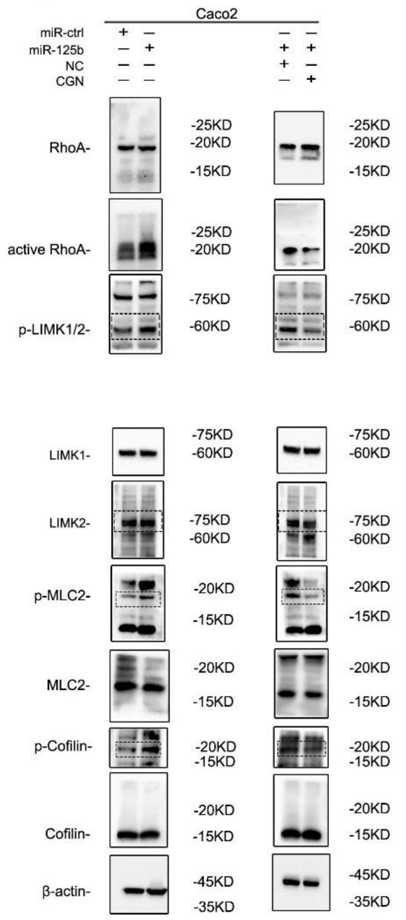

Figure S1B

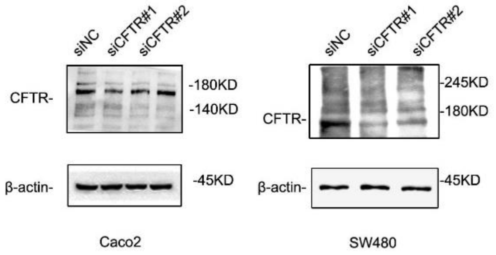

Figure S1C

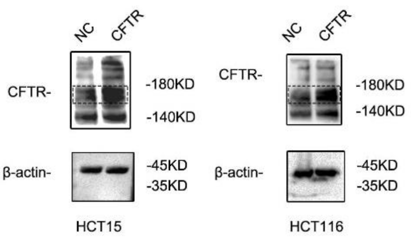

Figure S1D

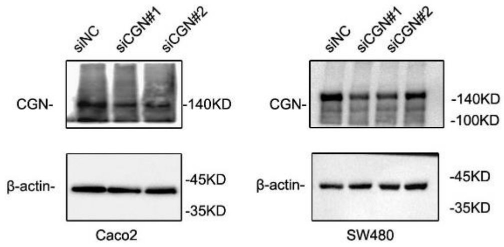

Figure S1E

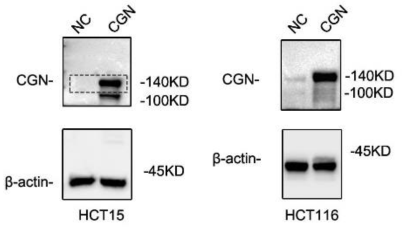

Supplement: Supplementary file 1 [file cancers-13-05710-s001.zip › Figure S2.pdf]
