# Supplementary figures and images for: miR-125b Promotes Colorectal Cancer Migration and Invasion by Dual-Targeting CFTR and CGN
Source: Cancers (Basel). 2021 Nov 15;13(22):5710. doi: 10.3390/cancers13225710 (PMC8616371; doi:10.3390/cancers13225710)

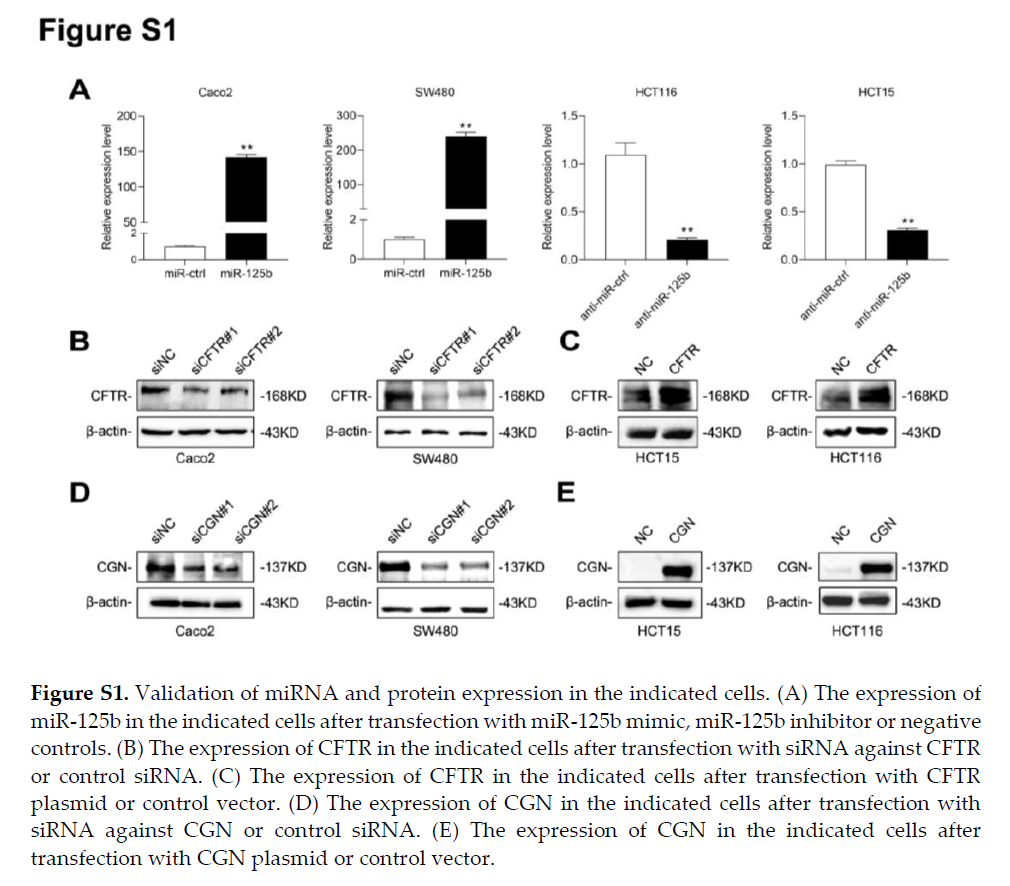

Supplement: Supplementary file 1 [file cancers-13-05710-s001.zip › Figure S1.png]
